# Transmission of Power by Electricity in Mining

**Published:** 1881-10

**Authors:** 


### TRANSMISSION OF POWER BY ELECTRICITY IN MINING.

The first instance on record of the application of electricity for the transmission of power is reported from France. M. Mathet has submitted the details to the Societie de l'Iindustrie Minerale. The St. Claude shaft at Blanzy was sunk to the depth of 500 metres (1,640 feet), for the purpose of searching for a faulted portion of the coal seams, and a heading was run from it across the strata. When this heading had reached a length of 400 metres (1,312 feet) the ventilation became so poor that the temperature at the face rose to 95 deg. Fah., and the miners could work only for a few hours. After some ineffectual attempts to improve the ventilation by simple means, it was decided to put in a fan 2.63 feet in diameter, and run it by power transmitted by electricity. An 8 to 10-horse power portable engine was put up above ground, and with it a Gramme dynamo-electric machine was run at a speed of 1,200 revolutions per minute. The electric current thus generated was conducted by a cable, consisting of seven 0.044 inch copper wires, to a second Gramme machine coupled directly with the fan, and placed in the heading near the shaft. Running at 700 to 800, it required 2½ horse power, the useful effect being at least 60 per cent. The temperature at the face was only lowered 5 deg., but the men could work in eight-hour shifts. The return current was conducted from the underground machine by an iron wire cable. The cost of the whole plant is stated to have been only one-third of what a machine for delivering compressed air to the heading would have required.—*Scientific American.*

